# Mind–body exercise and anxiety in middle-aged and older adults: a sequential mediation model of emotion regulation and sleep quality

**DOI:** 10.3389/fpubh.2026.1763761

**Published:** 2026-02-27

**Authors:** Yafei Zheng, Hengzhen Liang, Xiujie Ma

**Affiliations:** 1 The School of Affiliated Competitive Sports, Chengdu Sport University, Chengdu, China; 2 Physical Education Teaching and Research Office, Chengdu Experimental Primary School, Chengdu, China; 3 Chinese Guoshu Academy, Chengdu Sports University, Chengdu, China

**Keywords:** anxiety, chain mediation, emotion regulation, middle-aged and older adults, mind–body exercise, sleep quality

## Abstract

**Objective:**

This study examined the associations among mind–body exercise, emotion regulation, sleep quality, and anxiety in middle-aged and older adults, with particular attention to whether emotion regulation and sleep quality operate as sequential mediators linking mind–body exercise to anxiety.

**Methods:**

A cross-sectional survey was conducted with 382 middle-aged and older adults who regularly participated in mind–body exercise. Mind–body exercise, emotion regulation, sleep quality, and anxiety were assessed using validated self-report scales, including the Physical Activity Rating Scale (PARS-3) Questionnaire, the Emotion Regulation Questionnaire, the Pittsburgh Sleep Quality Index (PSQI), and the GAD-7. Data analyses were performed using SPSS 26.0 for descriptive statistics and correlations, and AMOS 24.0 for structural equation modeling to test the mediation and chain mediation pathways.

**Results:**

Mind–body exercise was significantly and negatively associated with anxiety (*p* < 0.01). Both emotion regulation and sleep quality served as significant mediators of this relationship. In addition, a sequential mediation pathway linking mind–body exercise, emotion regulation, sleep quality, and anxiety was supported, indicating that better emotion regulation was associated with improved sleep quality, which in turn was related to lower anxiety levels.

**Conclusion:**

These findings suggest that emotion regulation and sleep quality jointly help explain how mind–body exercise relates to anxiety in middle-aged and older adults. The results provide insight into the interconnected psychological processes linked with mind–body practices and highlight their potential value as non-pharmacological options for anxiety management in aging populations.

## 1 Introduction

Anxiety has become one of the most prevalent mental health concerns worldwide, impairing emotional functioning, disrupting cognitive processes, and contributing to sleep disturbances and various psychosomatic conditions. Its widespread impact poses a substantial public health challenge (1, 2). Conventional interventions such as pharmacotherapy and cognitive–behavioral therapy, while effective for many individuals, face limitations related to accessibility, side effects, and long-term adherence. These constraints have increased interest in safe, low-cost, and sustainable non-pharmacological approaches (3). Within this context, mind–body exercise—an umbrella term encompassing practices that integrate physical movement with breathing and attentional regulation—has received growing attention in relation to psychological well-being.

In China, mind–body practices such as Tai Chi and Qigong are deeply embedded in daily life and are widely practiced in community and public settings, particularly among middle-aged and older adults. This cultural embeddedness and widespread accessibility provide an important contextual background for examining the psychological correlates of mind–body exercise in this population.

Evidence from prior research suggests that practices such as Tai Chi, and Qigong are associated with reduced anxiety symptoms (4, 5). However, much of the existing literature has focused on direct associations, while the underlying psychological mechanisms remain insufficiently understood. Emotion regulation and sleep quality, both closely linked to anxiety, have been proposed as relevant psychological correlates. Mind–body exercise has been associated with greater emotional awareness and regulatory capacity, as well as reduced psychological tension (6), which may be linked to improvements in sleep quality—an important factor that, in turn, relates to lower anxiety levels (7, 8).

Despite these insights, prior studies have largely examined emotion regulation and sleep quality as independent mediators rather than as components of a sequential process. Whether enhancements in emotion regulation subsequently contribute to better sleep—and whether this sequence helps explain the association between mind–body exercise and anxiety—remains underexplored. Investigating this potential chain mediation pathway may advance theoretical understanding and offer more targeted evidence for anxiety management.

To address these gaps, the present study proposes a chain mediation model examining the associations among mind–body exercise, emotion regulation, sleep quality, and anxiety in middle-aged and older adults. By adopting a theory-informed, cross-sectional framework, this study seeks to extend existing literature and provide empirical evidence that may inform future longitudinal and intervention-based research on anxiety-related processes.

## 2 Literature reviews and hypotheses

### 2.1 The direct association between mind–body exercise and anxiety

Mind–body exercises (MBE), such as Tai Chi and Qigong, integrate slow movements, breathing regulation, and mindful attention to promote coordinated physiological and psychological functioning (9). Accumulating empirical evidence suggests that engagement in MBE is associated with lower anxiety levels across diverse populations and practice contexts (10). Studies examining Tai Chi, Qigong, and related mindfulness-oriented practices have consistently reported reductions in both state and trait anxiety following regular participation (11, 12).

Among middle-aged and older adults, regular engagement in MBE has likewise been associated with lower anxiety scores (4). Some findings even indicate that its short-term effects may approximate those of mild pharmacological or cognitive–behavioral interventions (13). Based on these observations, the present study proposes the following hypothesis:

*H*1: Mind-body exercise is negatively associated with anxiety among middle-aged and older adults.

### 2.2 The mediating role of emotion regulation

Emotion regulation (ER) refers to the processes through which individuals monitor, evaluate, and modify their emotional responses to achieve desired psychological outcomes (14). Increasing evidence suggests that ER plays an important mediating role in the association between mind–body exercise and anxiety among middle-aged and older adults. Mind–body exercises, through their emphasis on breath control, bodily awareness, and mindful attention, may support more adaptive emotional functioning in later life (15). Studies have shown that regular participation in practices such as Tai Chi and Qigong is associated with greater emotional clarity, reduced emotional suppression, and increased use of adaptive strategies such as cognitive reappraisal (16). Given the well-established link between impaired emotion regulation and heightened anxiety (17), improvements in ER may represent a key psychological pathway through which mind–body exercise is related to lower anxiety levels. Accordingly, the present study proposes the following hypothesis:

*H*2: Emotion regulation mediates the relationship between mind-body exercise and anxiety among middle-aged and older adults.

### 2.3 The mediating role of sleep quality

Sleep quality is a key indicator of physical and psychological well-being and is closely associated with anxiety, particularly among middle-aged and older adults who often experience age-related sleep disturbances (18). Previous studies have highlighted the reciprocal relationship between poor sleep and heightened anxiety, suggesting that sleep may serve as a crucial link in understanding mental health in later life (8).

Growing evidence indicates that mind–body exercises such as Tai Chi and Qigong are associated with improvements in sleep among older adults. These practices, through slow movement, breath regulation, and attentional focus, may promote relaxation and reduce cognitive and physiological arousal, thereby supporting better sleep continuity and satisfaction (19, 20). Given that better sleep quality is consistently linked to lower anxiety levels (21, 22), sleep quality may represent a psychological pathway through which mind–body exercise is associated with reduced anxiety. Therefore, the present study proposes the following hypothesis:

*H*3: Sleep quality mediates the relationship between mind-body exercise and anxiety among middle-aged and older adults.

### 2.4 The sequential mediating role of emotion regulation and sleep quality

Emerging evidence suggests that emotion regulation and sleep quality are not isolated mechanisms but form a dynamic, mutually reinforcing system that may jointly shape anxiety outcomes. Integrative reviews indicate that difficulties in regulating emotions are closely tied to disturbed sleep, while poor sleep in turn undermines regulatory capacity and increases emotional reactivity, creating a self-perpetuating cycle of vulnerability to anxiety and related problems (8). In older adults specifically, lower sleep quality has been linked to more negative affect and anxiety, highlighting sleep as a key pathway through which emotional functioning influences mental health in later life (23).

Recent studies have increasingly incorporated these processes into mediation and chain mediation frameworks. Research on physical activity has shown that better exercise engagement is associated with improved sleep quality, which in turn relates to lower anxiety levels (24). Other studies have applied chain mediation models to exercise-related outcomes, introducing emotion regulation ability and exercise adherence as sequential mediators between sleep quality and physical health indicators (25). These findings support the plausibility of modeling emotion regulation and sleep quality as interrelated mechanisms in the context of health behaviors. Against this background, mind–body exercise may be associated with more adaptive emotion regulation, which is further associated with sleep quality and lower levels of anxiety. Therefore, the present study proposes the following hypothesis:

*H*4: Emotion regulation and sleep quality sequentially mediate the relationship between mind-body exercise and anxiety among middle-aged and older adults.

### 2.5 Hypotheses and conceptual model

**Figure 1:**
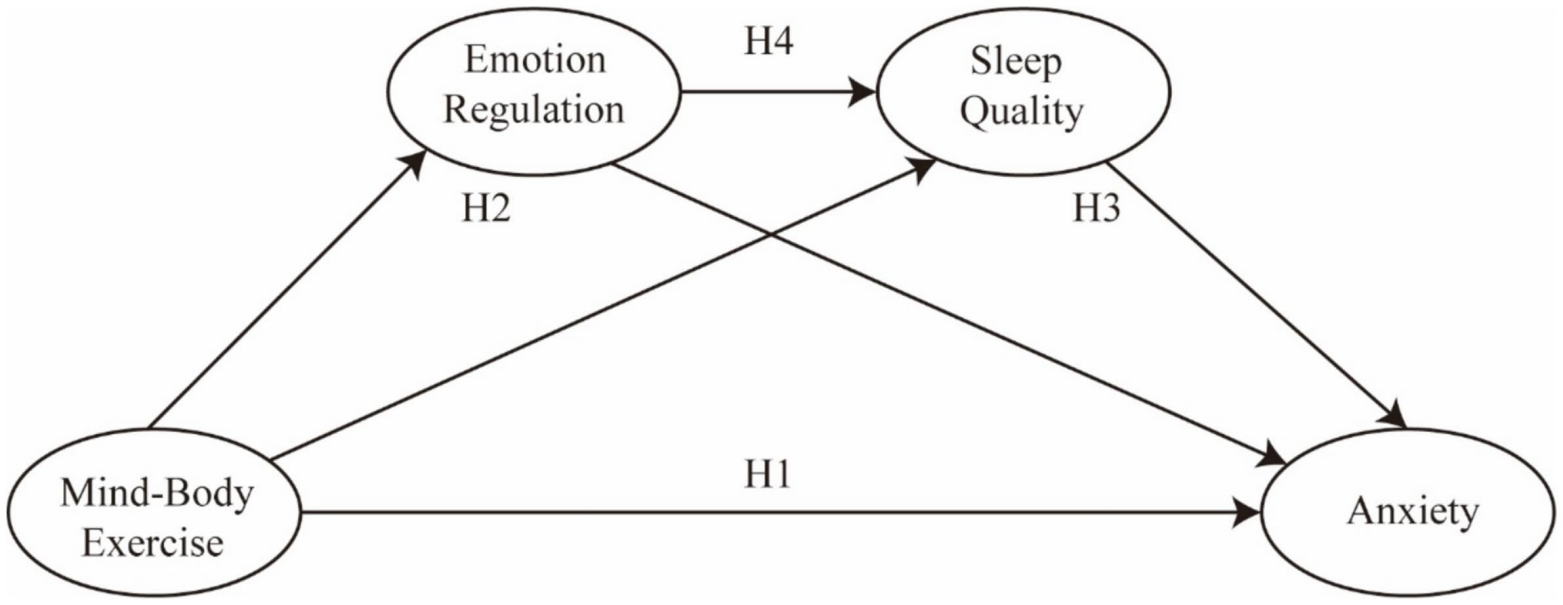
The hypothetical model.

As shown in Figure 1, this study proposes several hypotheses regarding the relationships among mind–body exercise, emotion regulation, sleep quality, and anxiety. First, mind–body exercise is assumed to be directly associated with anxiety (H1). Second, emotion regulation may serve as a mediating process between mind–body exercise and anxiety (H2). Third, sleep quality may also function as a mediator linking mind–body exercise to anxiety (H3). Finally, emotion regulation is expected to be positively associated with sleep quality (H4), forming a potential sequential pathway through which mind–body exercise may relate to different levels of anxiety.

## 3 Materials and methods

### 3.1 Participants and procedure

A combination of convenience sampling and snowball sampling was employed to recruit participants. As a result, the sample primarily consisted of individuals who were already engaged in mind–body exercise, which may introduce potential selection bias and limit the generalizability of the findings to the broader population. Eligible participants were middle-aged and older adults who had engaged in regular mind–body exercise—primarily low-intensity practices emphasizing mind–body harmony, such as Tai Chi and Qigong—at least once per week during the past 6 months and voluntarily agreed to participate. The inclusion criteria were as follows: (1) age ≥ 45 years; (2) ability to independently comprehend and complete the questionnaire; and (3) provision of written informed consent. Exclusion criteria included: (1) diagnosed severe cognitive impairment or neurological disorders (e.g., Alzheimer’s disease, Parkinson’s disease) that could interfere with questionnaire completion; (2) acute or chronic medical conditions limiting participation in physical activity; and (3) ongoing use of psychiatric medications or receipt of mental health treatment that could affect the assessment of emotional or anxiety-related symptoms. A total of 382 valid questionnaires were obtained.

Prior to data collection, researchers provided participants with a detailed explanation of the study’s purpose, content, and procedures. Participation was entirely voluntary and anonymous, and individuals were informed that they could withdraw at any time without consequences. The questionnaire consisted of demographic information, mind–body exercise participation, emotion regulation, sleep quality, and anxiety measures. To ensure the reliability and cross-cultural equivalence of the instruments, internationally validated standardized scales were used, and a forward–backward translation procedure was conducted to achieve semantic consistency. The study protocol was not pre-registered. Ethical approval for this study was obtained from the Research Ethics Committee of Chengdu Sport University. All procedures adhered to the principles of the Declaration of Helsinki.

### 3.2 Instruments

#### 3.2.1 Mind–body exercise

Mind–body exercise was assessed using the Physical Activity Rating Scale (PARS-3) developed by Liang (26). The PARS-3 is a concise and widely applied instrument in Chinese sport and health psychology research for quantifying individuals’ physical activity levels. It evaluates three core dimensions of exercise behavior—intensity, duration, and frequency—each rated on a five-point Likert scale, where higher scores indicate greater engagement in physical activity. In the present study, PARS-3 was employed to capture the behavioral level of exercise participation among individuals who regularly engaged in mind–body practices, rather than to assess the defining psychological characteristics of mind–body exercise, such as mindfulness, breath regulation, or mind–body integration. This operationalization is consistent with prior research that has applied PARS-3 to characterize exercise participation intensity in samples engaged in mind–body activities. Numerous empirical studies have confirmed its good reliability and validity in assessing exercise behavior and its psychological correlates in this population (27, 28).

#### 3.2.2 Emotion regulation

Emotional regulation refers to the process by which individuals influence their emotional responses through cognitive or behavioral strategies when faced with environmental stimuli and internal experiences (14). This study used the Emotion Regulation Questionnaire (ERQ) to measure this variable (29). This scale consists of two core dimensions: cognitive reappraisal and expressive suppression, with a total of 10 items, all rated using a 7-point Likert scale (1 = strongly disagree, 7 = strongly agree). Cognitive reappraisal reflects an individual’s ability to change emotional experiences by reinterpreting situations, while expressive inhibition measures an individual’s tendency to suppress external emotional expression. Previous studies have shown that ERQ has good reliability and validity in cross-cultural environments and is widely used in sports, health, and clinical psychology research (30, 31).

#### 3.2.3 Sleep quality

Sleep quality is a measure of an individual’s overall subjective perception of the process of falling asleep, maintaining sleep, and restorative aspects (32). This study used the Pittsburgh Sleep Quality Index (PSQI) to measure sleep quality. PSQI consists of 19 items covering 7 dimensions: subjective sleep quality, falling asleep time, sleep duration, sleep efficiency, sleep disorders, hypnotic drug use, and daytime dysfunction. According to the standard scoring procedure, each component is scored from 0 to 3, yielding a global score ranging from 0 to 21, with higher scores indicating poorer sleep quality. This scale has been widely used globally and has shown good reliability and validity in studies on sports populations and mental health (22).

#### 3.2.4 Anxiety

This study assessed anxiety using the Generalized Anxiety Disorder Scale-7 (GAD-7) developed by Spitzer et al. (33). The GAD-7 consists of 7 items, each rated on a 4-point Likert scale (0 = not at all, 1 = several days, 2 = more than half the days, 3 = nearly every day). The total score ranges from 0 to 21, with higher scores indicating more severe anxiety symptoms. Cutoff points of 5, 10, and 15 indicate mild, moderate, and severe anxiety, respectively. The GAD-7 has demonstrated excellent reliability, unidimensional structure, and strong criterion validity across general, clinical, and older adult populations (34). It has also been widely applied in studies on physical activity, sleep, and mental health.

### 3.3 Control variables

To address potential confounding influences, a set of demographic and socioeconomic covariates were included based on established theoretical and empirical evidence linking these factors to sleep quality, anxiety, and psychological well-being among middle-aged and older adults. Age and gender were controlled because both are consistently associated with age-related changes in sleep patterns, emotional regulation, and anxiety prevalence in later life (35, 36). Marital status was included as a proxy for social support and living context, which are known to influence emotional well-being and sleep quality (37). Education level and monthly income were controlled as indicators of socioeconomic status, as these factors are closely related to health literacy, stress exposure, access to health-promoting resources, and engagement in physical activity (38, 39). Together, these variables represent widely recognized background characteristics that may systematically influence both mind–body exercise participation and mental health outcomes in older populations. Although other potentially relevant clinical and behavioral factors may also contribute to anxiety, such variables were not assessed in the present study and therefore could not be included as covariates.

### 3.4 Analysis

All analyses were conducted using IBM SPSS 26.0 and AMOS 24.0. Prior to analysis, data were screened for missing values, outliers, and normality. Descriptive statistics and Pearson correlations were computed for all study variables. To assess potential common method bias, Harman’s single-factor test was performed. The first unrotated factor accounted for less than 40% (38.94%) of the total variance, indicating that common method bias was not a serious concern.

Internal consistency reliability was evaluated using Cronbach’s alpha. Confirmatory factor analyses (CFAs) were then conducted in AMOS to examine the measurement properties of emotion regulation, sleep quality, and anxiety. Model fit was assessed using χ^2^/df, CFI, TLI, CFI, NFI, IFI, RMSEA, and SRMR.

The hypothesized serial mediation model was tested using structural equation modeling (SEM) in AMOS. Mind–body exercise was specified as the independent variable, emotion regulation and sleep quality as sequential mediators, and anxiety as the dependent variable. Indirect effects were estimated using bias-corrected bootstrapping with 5,000 resamples, and significance was determined by 95% confidence intervals that did not include zero.

Age, gender, marital status, education level, and monthly income were included as control variables and were specified as predictors of the mediators and outcome. Statistical significance was set at *p* < 0.05.

## 4 Results

### 4.1 Descriptive statistics and correlations among the main study variables

Table 1 presents the demographic characteristics of the 382 participants. The majority were female, aged between 45 and 64 years, and most were married. In terms of education, the sample was relatively diverse, though junior high school was the most common level attained. The majority reported a monthly income between 1,001 and 3,000 RMB.

**Table 1:** Demographic characteristics of the samples (*N* = 382).

Variable	Category	Frequency (n)	Percentage (%)
Gender	Male	146	38.22%
	Female	236	61.78%
Age	45–54	136	35.60%
	55–64	143	37.44%
	65–74	79	20.68%
	>75	24	6.28%
Marital status	Married	354	92.67%
	Divorced/Single	11	2.88%
	Widowed	17	4.45%
Education level	Primary school or below	75	19.63%
	Junior high school	118	30.89%
	High school	104	27.23%
	College or above	85	22.25%
Monthly income(RMB)	<1,000	91	23.82%
	1,001-3,000	216	56.55%
	3,001-5,000	62	16.23%
	>5,000	13	3.40%

Table 2 presents the means, standard deviations, and Pearson correlations among the study variables. The mean scores ranged from 1.19 to 4.07, with standard deviations between 0.90 and 1.04, indicating moderate variability. Mind–body exercise was positively associated with emotion regulation and sleep quality, anxiety showed significant negative correlations with all three variables. Importantly, no bivariate correlation exceeded the threshold of 0.85, and all VIF values were within acceptable limits, suggesting no concerns regarding multicollinearity.

**Table 2:** Descriptive statistics and correlations among primary variables.

Variable	M	SD	1	2	3	4
1 Mind–body exercise	1.19	0.90	1			
2 Emotion regulation	4.07	1.04	0.409**	1		
3 Sleep quality	2.60	1.01	0.371**	0.394**	1	
4 Anxiety	2.67	1.03	−0.393**	−0.470**	−0.428**	1

*p < 0.05, **p < 0.01 (two-tailed).

### 4.2 The test of reliability and validity

Table 3 summarizes the reliability and convergent validity indices for the primary constructs. All four variables demonstrated adequate internal consistency, with Cronbach’s *α* coefficients ranging from 0.809 to 0.933, exceeding the commonly recommended threshold of 0.70. Composite reliability (CR) values were similarly high (0.811–0.933), indicating strong construct reliability. The average variance extracted (AVE) values ranged from 0.551 to 0.667, all above the 0.50 criterion, suggesting that each construct explains a substantial proportion of variance in its observed indicators (40). Overall, the results provide evidence of good reliability and convergent validity for all measurement scales used in this study.

**Table 3:** Validity and reliability tests of the questionnaires.

Variable	Cronbach’s α	CR	AVE
Mind–body exercise	0.809	0.811	0.588
Emotion regulation	0.924	0.924	0.551
Sleep quality	0.928	0.928	0.648
Anxiety	0.933	0.933	0.667

CR, composite reliability; AVE, average variance extracted.

In addition, the overall model fit was evaluated using multiple fit indices, including χ^2^/df, GFI, TLI, CFI, NFI, IFI, SRMR and RMSEA. As shown in Table 4, all fit indices exceeded the commonly recommended threshold of 0.80, with GFI = 0.942, TLI = 0.998, CFI = 0.998, NFI = 0.951, and IFI = 0.969, indicating a good model fit. The SRMR value was 0.031, RMSEA value was 0.010, which is well below the conventional cutoff value of 0.08, suggesting a close and acceptable fit between the hypothesized model and the observed data (50).

**Table 4:** Model fit indices for the measurement model.

	χ^2^/df	GFI	TLI	CFI	NFI	IFI	SRMR	RMSEA [90%CI]
Indices	1.034	0.942	0.998	0.998	0.951	0.969	0.031	0.010 [0.00, 0.02]

GFI, Goodness of Fit Index; RFI, Relative Fit Index; CFI, Comparative Fit Index; NFI, Normed Fit Index; IFI, Incremental Fit Index; SRMR, Standardized Root Mean Square Residual; RMSEA, Root Mean Square Error of Approximation.

### 4.3 The chain mediation model analysis

**Figure 2:**
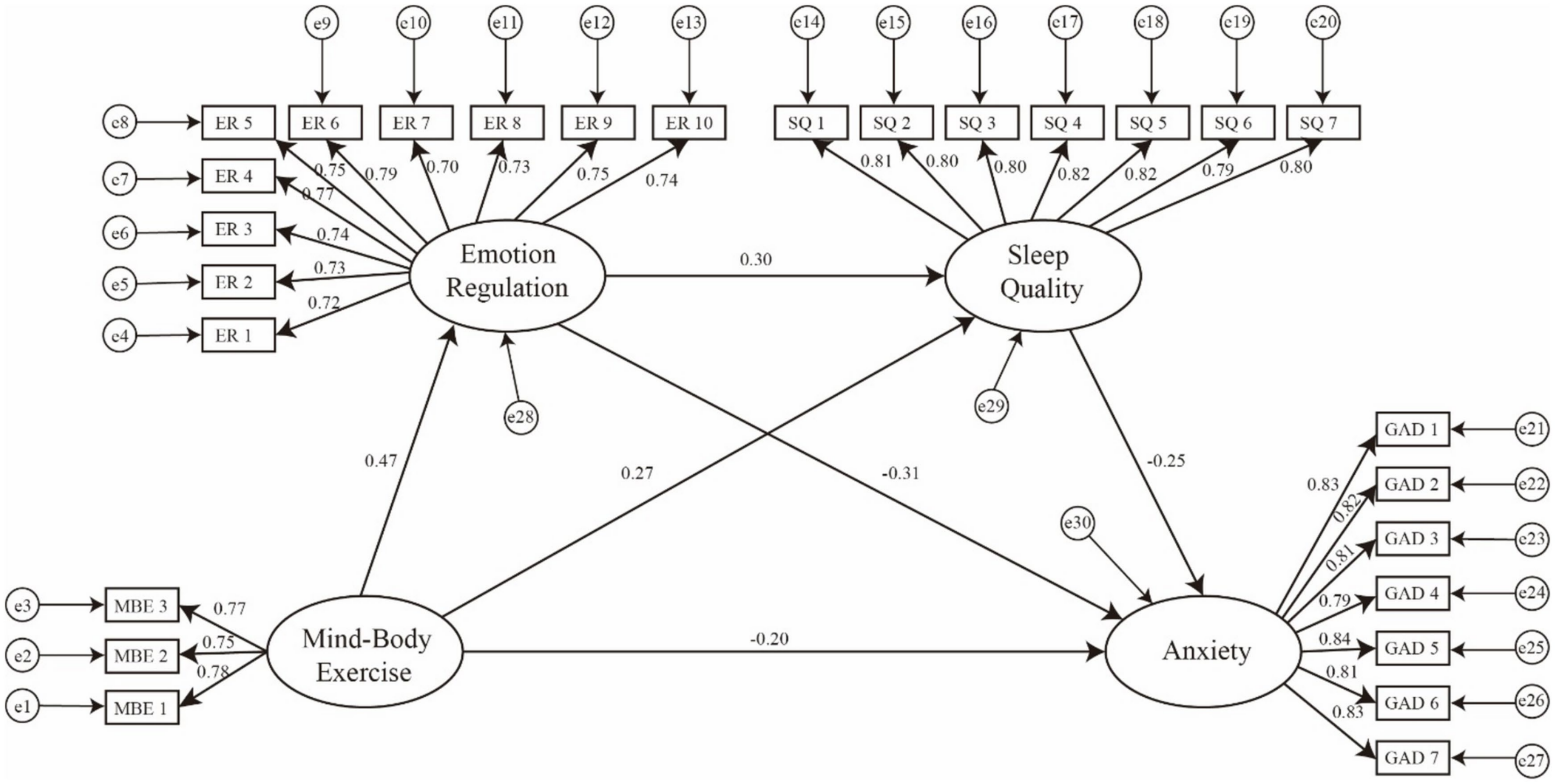
Chain mediation model illustrating the relationships among variables. Values represent standardized regression coefficients (*β*). Unstandardized coefficients are reported in Table 5.

To further test the hypothesized chain mediating model, structural equation modeling was conducted in AMOS with bias-corrected bootstrap estimation (5,000 resamples). As shown in Table 5 and Figure 2, mind–body exercise significantly and negatively predicted anxiety (direct effect: B = −0.164, 95% CI [−0.273, −0.061]), supporting the direct effect hypothesis.

**Table 5:** Bootstrap analysis of chain mediating effect.

	Effect	BootSE	BootLLCI	BootULCI
Indirect 1: MBE → ER → GAD	−0.114**	0.025	−0.166	−0.068
Indirect 2: MBE → SQ → GAD	−0.053**	0.020	−0.098	−0.021
Indirect 3: MBE → ER → SQ → GAD	−0.028**	0.009	−0.047	−0.012
Indirect effect	−0.195**	0.031	−0.262	−0.138
Direct effect	−0.164**	0.054	−0.273	−0.061
Total effect	−0.630**	0.048	−0.455	−0.268

MBE, mind–body exercise; ER, emotion regulation; SQ, sleep quality; GAD, anxiety. All effects are reported as unstandardized coefficients (B). BootSE, BootLLCI, and BootULCI represent the bootstrap standard error and the lower and upper bounds of the 95% bias-corrected bootstrap confidence intervals, respectively. Standardized coefficients are presented in Figure 2 for illustrative purposes. **p < 0.01.

Regarding the indirect pathways, the results indicated three significant mediating routes. First, mind–body exercise indirectly reduced anxiety through emotion regulation (Indirect 1: B = −0.114, BootCI [−0.166, −0.068]), supporting the mediation hypothesis for emotion regulation. Second, mind–body exercise indirectly predicted lower anxiety via sleep quality (Indirect 2: B = −0.053, BootCI [−0.098, −0.021]). Third, the sequential pathway linking mind–body exercise → emotion regulation → sleep quality → anxiety was also significant (Indirect 3: B = −0.028, BootCI [−0.047, −0.012]), providing evidence for the proposed chain mediation mechanism.

The total indirect effect was statistically significant (B = −0.195, BootCI [−0.262, −0.138]), and the overall total effect remained significant (B = −0.630, BootCI [−0.455, −0.268]), indicating that both independent and sequential mediating processes contribute to the relationship between mind–body exercise and anxiety.

In addition, Figure 2 presents the structural model with standardized path coefficients illustrating the mediating roles of emotion regulation and sleep quality in the relationship between mind–body exercise and anxiety. As shown in the figure, mind–body exercise was positively associated with emotion regulation (*β* = 0.47) and sleep quality (*β* = 0.27), while showing a negative association with anxiety (*β* = −0.20). Emotion regulation was positively associated with sleep quality (*β* = 0.30) and negatively associated with anxiety (*β* = −0.31). Sleep quality was also negatively related to anxiety (*β* = −0.25).

All measurement indicators demonstrated satisfactory standardized factor loadings (0.72–0.84), indicating acceptable convergent validity. Collectively, the structural paths support the hypothesized chain mediation framework, suggesting that mind–body exercise may be associated with anxiety both directly and indirectly through emotion regulation and sleep quality.

## 5 Discussion

### 5.1 The direct association between mind–body exercise and anxiety

The present study identified a significant negative association between mind–body exercise and anxiety, indicating that individuals who engage more frequently in such practices tend to report lower anxiety levels. This pattern is consistent with previous findings showing that mind–body practices—such as Tai Chi, Qigong, and Baduanjin—are related to improved emotional well-being and reduced physiological tension (41, 42). Mind–body exercise emphasizes gentle movements, rhythmic breathing, and focused attention, which may be associated with greater interoceptive awareness and may be related to better management of physiological and emotional responses (43). These characteristics may help contextualize its association with lower levels of anxiety observed in this study.

Beyond social engagement, mind–body exercise may also be associated with anxiety through non-social processes related to bodily awareness and self-regulation. Prior research suggests that the coordinated use of movement and breathing in mind–body practices may be linked to reduced subjective arousal and a calmer perception of internal bodily states, which are psychosocial factors relevant to anxiety (44, 45). These processes are not directly examined in the present study but may represent complementary pathways discussed in the broader literature.

In addition, mind–body practices are often performed in parks or community-based settings, which may provide opportunities for social interaction and emotional exchange. Such social engagement has been associated with lower perceived loneliness and better emotional well-being, factors that are themselves related to lower anxiety. Taken together, mind–body exercise may be interpreted not only as a form of physical activity, but also as a socially embedded practice whose combined bodily, attentional, and social characteristics are associated with more favorable psychological outcomes among middle-aged and older adults.

### 5.2 The mediating role of emotion regulation

The findings of this study suggest that emotion regulation may function as a mediating role in the relationship between mind–body exercise and anxiety. This pattern of associations may be interpreted in relation to cognitive, behavioral, and psychological processes. At the cognitive level, mind–body practices such as Tai Chi and Qigong emphasize the coordination of movement, breathing, and bodily sensations, creating a mindfulness-like state that may be associated with greater awareness and acceptance of emotional experiences (46). The philosophical foundation of these practices, including ideas such as “harmonizing with nature,” may also encourage individuals to adopt a less resistant and more receptive attitude toward negative emotions.

From a behavioral perspective, the low-intensity and non-competitive nature of mind–body exercise makes it a sustainable activity, and the sense of control or self-efficacy experienced during regular practice may be related to emotion regulation, with greater willingness to engage in adaptive emotional strategies. Psychologically, mind–body exercise highlights the reconnection between physical states and emotional experiences, which may help counteract the sense of disconnection often reported by individuals with heightened anxiety. The calmness and focused attention elicited during practice may accumulate as positive emotional experiences, being associated with greater psychological resilience as well as more effective emotion regulation.

### 5.3 The mediating role of sleep quality

The findings of this study suggest that sleep quality may play a mediating role in the association between mind–body exercise and anxiety, a pattern that can be interpreted in relation to psychological and behavioral processes. High-quality, restorative sleep is typically associated with greater emotional stability, fewer mood fluctuations, and more effective emotion regulation (47). Individuals who report better sleep tend to report clearer thinking and a more balanced emotional state when encountering stress, characteristics that are commonly associated with lower anxiety levels. Sleep quality is also closely related to cognitive processing styles. Those with adequate sleep generally tend to report stronger attentional control, greater cognitive flexibility, and more adaptive problem-solving abilities (48), which are associated with lower susceptibility to worry, rumination, or other anxiety-related cognitive tendencies.

In addition, positive sleep experiences may be associated with greater motivation to maintain regular engagement in mind–body exercise and with the development of more stable, health-oriented behavioral routines. Taken together, these patterns suggest that sleep quality may serve as a relevant psychological and behavioral correlate through which mind–body exercise is associated with lower levels of anxiety, rather than as an isolated or independent mechanism.

### 5.4 The sequential mediating role of emotion regulation and sleep quality

The findings of this study suggest a sequential pattern of associations linking mind–body exercise and anxiety through emotion regulation and sleep quality. This pattern provides an integrative perspective for understanding how multiple psychological processes may operate in conjunction rather than in isolation. Individuals with higher emotion regulation capacities tend to manage emotional responses more effectively, which may be associated with a psychological state more conducive to consistent and restorative sleep (8). And better sleep quality is typically associated with more stable emotional experiences, clearer cognitive processing, and fewer anxiety-related thought patterns (49).

This sequence of relationships suggests that emotion regulation and sleep quality are not independent processes but may be conceptually complementary. Enhanced emotion regulation may be associated with better sleep quality, while high-quality sleep may also be related to greater emotional management capacity. Together, they represent an important set of psychological correlates linking mind–body exercise and anxiety. This sequential mediation framework highlights that when explaining the relationship between physical and mental exercise and mental health, it is necessary to consider the interaction of multiple psychological systems rather than viewing them as a single, independent mechanism.

It is also important to distinguish between statistical significance and practical or clinical significance when interpreting these findings. Although the sequential mediation effect reached statistical significance, its magnitude was modest. Accordingly, the results should not be interpreted as evidence of direct clinical efficacy. Instead, they suggest that mind–body exercise may be meaningfully associated with anxiety at the population or behavioral level, particularly through its links with emotion regulation and sleep quality. From a public health perspective, even modest indirect effects may have practical relevance when considered across large populations or when incorporated into broader, multifaceted health promotion strategies.

### 5.5 Practical implication

The findings of this study offer several practical implications for promoting mental well-being among middle-aged and older adults. First, mind–body exercise may represent an accessible and low-cost behavioral option that can be considered within community-based health promotion programs. Its emphasis on slow movement, breath regulation, and attentional focus suggests that such practices may be particularly suitable for individuals who are less inclined or able to engage in high-intensity physical activity.

Second, the observed associations involving emotion regulation and sleep quality highlight two interrelated psychological domains that may be relevant when designing or implementing mind–body–based programs. Interventions that incorporate elements aimed at enhancing emotional awareness, acceptance, and adaptive coping may align with the psychological processes associated with mind–body exercise. Similarly, integrating sleep education or sleep-supportive behavioral routines alongside mind–body practice may be consistent with a more comprehensive approach to anxiety-related well-being.

Finally, the social and communal nature of many mind–body activities suggests that group-based formats may facilitate participation, foster social connectedness, and support broader psychological well-being. Taken together, these practical considerations underscore the potential value of mind–body exercise when viewed within a multidimensional framework that integrates physical activity, psychological processes, and social context, rather than as a single or isolated intervention.

### 5.6 Limitations

Several limitations should be acknowledged when interpreting the findings of this study. First, the cross-sectional design does not allow for causal inferences regarding the relationships among mind–body exercise, emotion regulation, sleep quality, and anxiety. Longitudinal or experimental studies are needed to clarify the temporal ordering of these variables. Second, all measures were based on self-report questionnaires, which may be subject to recall bias, shared method variance, and social desirability effects. Future research could incorporate objective indicators of sleep or behavioral measures of emotion regulation to strengthen the validity of the findings. Third, although the study focused on middle-aged and older adults, the sample was limited to a specific cultural and geographic context, which may restrict the generalizability of the results. Replication in more diverse populations would be beneficial. Fourth, even within the correlational framework adopted in this study, although several key demographic and socioeconomic covariates were controlled, residual confounding related to unmeasured clinical conditions, medication use, and detailed exercise history cannot be ruled out, which may weaken the internal validity of the observed associations. Finally, the present model examined only emotion regulation and sleep quality as mediators; other relevant psychological processes—such as mindfulness, social connectedness, or stress appraisal—may also play a role. Future work could test more comprehensive models to provide a broader understanding of how mind–body exercise relates to mental well-being.

## 6 Conclusion

This study examined the relationships among mind–body exercise, emotion regulation, sleep quality, and anxiety in middle-aged and older adults. The findings suggest that mind–body exercise is associated with lower anxiety both independently and in association with emotion regulation and sleep quality, with a sequential pattern of associations further linking these processes. These results underscore the relevance of mind–body exercise as a multidimensional health practice that relates to emotional and behavioral factors relevant to psychological well-being. Future research using longitudinal or experimental designs is warranted to validate these pathways and deepen understanding of how mind–body practices support mental health in aging populations.

## Data Availability

The raw data supporting the conclusions of this article will be made available by the authors, without undue reservation.
